# Dynamic development of syntactic complexity in second language writing: A longitudinal case study of a young Chinese EFL learner

**DOI:** 10.3389/fpsyg.2022.974481

**Published:** 2022-08-09

**Authors:** Zhihong Wang

**Affiliations:** College of Foreign Languages, Ocean University of China, Qingdao, China

**Keywords:** L2 writing, syntactic complexity, dynamic development, interaction, young learner

## Abstract

The present study analyzes the English diaries written by a young Chinese English as a foreign language (EFL) learner over a 1-year period in an attempt to determine the developmental process of Chinese EFL young learners’ written language in terms of syntactic complexity. This study aimed to use a wide range of metrics to explore densely collected data based on Dynamic Systems Theory. The longitudinal study data were analyzed through eight large-grained measures related to sentential, clausal, and phrasal features by using L2 Syntactic Complexity Analyzer, as well as fine-grained measures related to seven modifiers, an adjective placed before a noun, ordinal numerals are indicated (ADJA), cardinal numeral (CARD), predicative adjective, adverbial adjective (ADJP), adverbs (ADV), prepositional phrases (PREP), subordinate (SUB), and relative clause (REL), through manual annotations. The results show that, first, the developmental process was not linear but spiral in terms of large-grained measures. The sentential level varied, and the development process of the young learner is different from Chinese English learners studying in colleges. Second, the dynamic features of fine-grained measures are also prominent in the seven indicators. ADJA, PREP, and REL showed a steady increase, ADJP showed an obvious decline, while ADV and SUB first increased and then declined. Third, the correlation analysis revealed a competitive interaction within fine-grained measures and between large-grained and fine-grained measures.

## Introduction

L2 syntactic complexity and its development over time have been studied by adopting a Dynamic Systems Theory (DST) approach in recent years. DST works at the system level and explains the system’s behavior, not at the elemental level (Larsen-Freeman and Cameron, 2008). From this perspective, language development is considered a complex system which contains many subsystems that interact, are interconnected, and are interdependent. Researchers have been trying to explore the development and variations of learners’ language production from a multi-dimensional perspective by analyzing different variables that support or compete with each other to discover the factors that influence L2 development (e.g., Larsen-Freeman, 2006; Verspoor et al., 2008, 2012; Vyatkina et al., 2015). Bulté and Housen (2014) have stated that complexity can be viewed as an index of language development and progress, being treated as a valid and basic descriptor of second language performance in L2 research. It is also suggested that more longitudinal investigations of complexity measures are needed to obtain a full view of developmental trajectories (Ortega and Byrnes, 2008). The present study focuses on syntactic complexity as defined as “the degree of variation, sophistication, and elaboration of the syntactic structures used in language production” (Lu et al., 2020).

Adolescents constitute the main body of beginner and intermediate learners and are surely different from adult learners in their cognition and emotions. Therefore, it is significant to study L2 developmental process of adolescent learners. However, to the best of my knowledge, empirical studies on the development in writing of L2 young learners based on DST are rare. Therefore, in the present study I use several large-grained and fine-grained syntactic complexity measures to investigate an individual young learner’s development in writing by tracking dense naturalist data of her 365 diary written entries to explore (i) how syntactic complexity develops in writing over time (ii) how developmental paths measured by different metrics correlate with each other, and (iii) what measures can capture the writing development of the young learner.

## Methods

### Participant

This is a longitudinal case study of a Chinese girl named Alice aged 11 at the time of the study. She had passed her third-grade and had completed 2-week fourth grade study in an elementary school of the United States from August 2014 to August 2015. She had learned the alphabet and some basic spoken English used in daily life before going abroad. She was admitted to ESL classes at the beginning of the first half of the school year (September to February before spring break) in the United States. After that, she was made to join the school’s regular third-grade class for 5 months until the summer vacation started; she did her regular coursework and homework. At the end of the school year, Alice passed all her third-grade tests at the school. She had started keeping a daily English diary at the suggestion of her parents in order to improve her English level as she had returned to China in 2016, and continued doing so until 2018, before she entered junior high school.

### Metrics

#### Large-grained measures

With respect to the syntactic complexity measures mentioned and established by earlier researchers, the present study follows Norris and Ortega (2009) and other recommendations reported in the literature (e.g., Bulté and Housen, 2014; Yang et al., 2015; Yoon and Polio, 2017) to examine syntactic complexity as a multi-dimensional construct. Therefore, a few syntactic complexity measures were selected for this study based on Lu (2011) to gauge the complexity of syntactic organization by L2 Syntactic Complexity Analyzer (hereafter L2SCA; Lu, 2010) at the sentential, clausal and phrasal levels (refer to Table 1).

**TABLE 1:** Summary of the large-grained measures and fine-grained measures.

	Level	Measure	Definition
	Sentential	MLS	Mean Length of Sentence
		MLT	Mean Length of T-Unit
		MLC	Mean Length of Clause
Large-Grained measures	Clausal	T/S	Number of T-units per sentence
		DC/T	Number of dependent clause per T-unit
		C/T	Number of clauses per T-unit
	Phrasal	CP/C	Number of coordinate phrases per clause
		CN/C	Number of complex noun phrases per clause
Fine-grained measures	Lexical modifiers	ADJA	An adjective placed before a noun, ordinal numerals are indicated
		CARD	Cardinal Numeral
		ADJP	Predicative adjective, adverbial adjective
		ADV	Adverbs
	Phrasal	PREP	Prepositional phrases
	Subordinate	SUB	Adverbial clause
	Clause Modifiers	REL	Relative clause

Three sets of measures on sentential syntactic complexity were chosen: Mean Length of Sentence (MLS), Mean Length of T-Unit (MLT), and Mean Length of Clause (MLC). They represent a different but interrelated aspect of sentence complexity. MLS indicates the overall sentence complexity; MLT indicates overall T-unit complexity; and MLC indicates the elaboration at the clause level (Yang et al., 2015).

Three sets of measures on clausal syntactic complexity were chosen: T-Units per Sentence (T/S); Dependent Clauses per T-Unit (DC/T); and Number of Clauses per T-Unit (C/T), which pertain to clausal coordination, subordination, and embedding.

Two sets of measures of phrasal syntactic complexity were chosen: Coordinate Phrases per T-Unit (CP/T) and Complex Noun Phrases per T-Unit (CN/T). These pertain to phrasal coordination and noun phrase complexity.

#### Fine-grained measures

Besides the large-grained measures, several studies have recommended that much narrower measures are needed to capture L2 learners’ writing development (e.g., Biber et al., 2011; Mazgutova and Kormos, 2015). The approach of using fine-grained measures is helpful in understanding the types of detailed structures learners expand in the sentences (Kyle and Crossley, 2018). Jiang et al. (2019) found that usage of different modifiers can serve as developmental indicators for the young beginner learners progressing from pre-attributive modifiers to post-phrasal and clausal modifiers. Vyatkina et al. (2015) investigated the written output in the writing of English of beginner learners of German using seven indicators an adjective placed before a noun, ordinal numerals are indicated (ADJA), cardinal numeral (CARD), predicative adjective, adverbial adjective (ADJP), adverbs (ADV), prepositional phrases (PREP), subordinate (SUB), and relative clause (REL). Considering the similarity between the German and English language, and as both belong to the Indo-European language family, the fine-grained indicators in the present study are drawn from those used by Vyatkina et al. (2015; see Table 1, refer to detail examples in Supplementary material). First are lexical modifiers (cardinal, adjective, predicate adjective, and adverb), second are phrasal modifiers (prepositional phrases) headed by a specific part of speech, and third are subordinate modifiers that are attached to a sentence (adverbial clause) or noun (relative clause). According to Vyatkina et al. (2015), these types of modifiers can be included in the basic list of grammatical modifiers.

### Data collection and analysis

I collected the English diaries written by Alice over a 1-year period in 2017 one and a half year later after she came back from the United States. The diaries were input into electronic form and comprised 20,665 words. A corpus was then established (refer to corpus details in Supplementary material). The daily diary represented the completely natural English output spontaneously written without any time limit, without any given topic, and word limit.

L2SCA requires that the English written output be analyzed in a plain text file with no annotated information other than the essay itself. Since each diary contained different numbers of words, including some short entries that did not meet the minimum number of words (50 words) that L2SCA requires, on the entries for each 7-day period of the diary were combined into one file, which was named based on the time it was written. For example from 1 to 7 January 2017, seven diary texts were synthesized into one named 2017 week1.txt; from 8 to 15 January 2017, the diary texts were synthesized and named 2017 week2.txt; and so on. The text of the last week included eight entries from 24 to 31 December, named 2017 week52.txt. There were 52 texts in total. Each of the named text files was put into a different folder and analyzed by L2SCA. Eight syntactic complexity indices for each written text file were obtained and an Excel file of the data was prepared. The development trajectories of the indicators were described with the help of the trend line in Excel based on the 52-week syntactic complexity data and summarized after comparing the quantitative results of the different stages. The seven types of modifiers in the corpus were manually annotated by two English instructors. The interrater reliability was measured by using Cohen’s kappa, which had the value 0.91, and the discrepancies between their annotations were resolved through discussions.

## Results

### Large-grained measures

**FIGURE 1:**
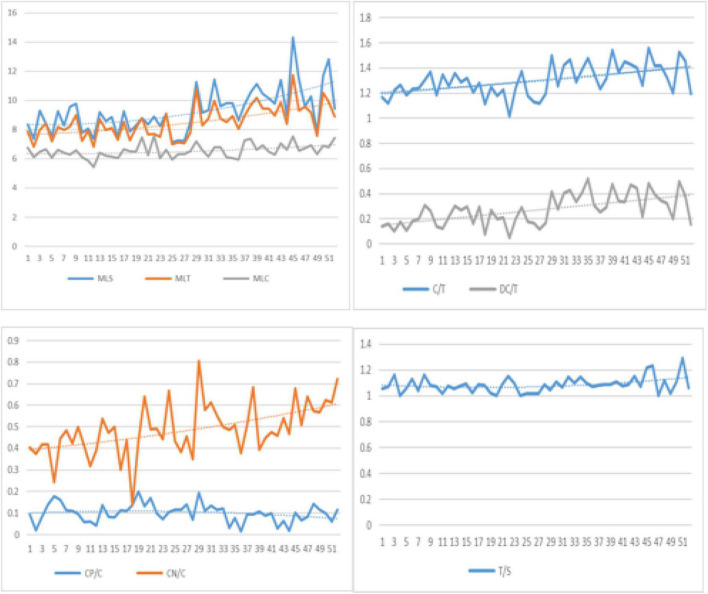
The development of eight large-grained measures over time.

**FIGURE 2:**
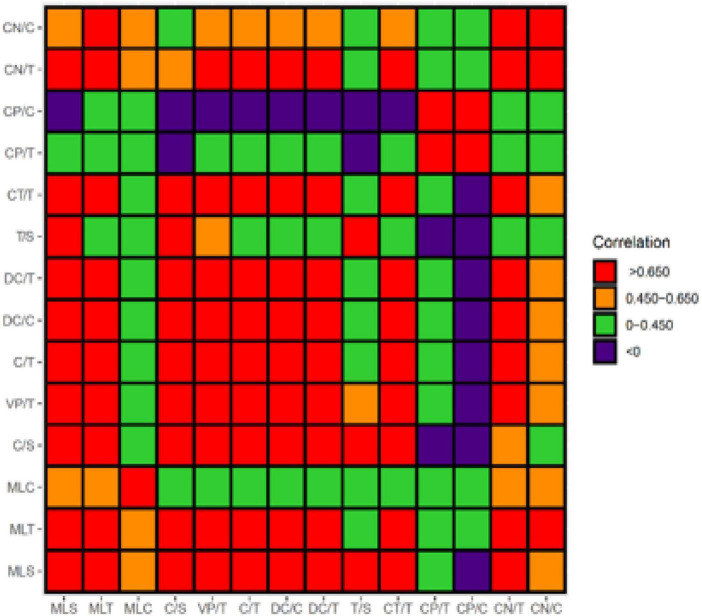
Correlation among syntactic complexity indicators. According to Wolfe-Quintero et al. (1998), correlation greater than 0.650 is a strong correlation, 0.450–0.650 is a moderate correlation, and less than 0.450 is a weak correlation.

The eight measures of syntactic complexity indicate that the changes in both peak and regression were unstable during the process of development (see Figure 1). The graph representing the growth and decline trends are curved, and reflect obvious dynamic characteristics. The increase in MLS and MLT was obvious, while a slight increase in the MLC during the development process was observed. C/T and DC/T all showed an upward trend while T/S was relatively stable. CP/C and CN/C of the phrasal structure fluctuated and exhibited an opposite developmental trend. I also generated a heat map using R analysis to conduct clustering correlation analysis on all the indicators (see Figure 2). In the heat map, the value generated by calculating the correlation between each two measures is represented by a certain color in a matrix. A heat map depicts the strength of the relationship between measures using different colors. Just like the frequency distribution of the spectrum, as labeled, the redder the color, the stronger the positive correlation; the more purple the color, the stronger the negative correlation; orange and green are less correlated than red. A strong correlation was found between MLS and MLT, and a strong correlation between C/T index with MLS, MLT, DC/T, and T/S was revealed. CP/C and the other indices including MLS, C/T, DC/T, T/S, and CN/C correlated negatively, indicative of a state of competition between them in the development process.

### Fine-grained measures

**FIGURE 3:**
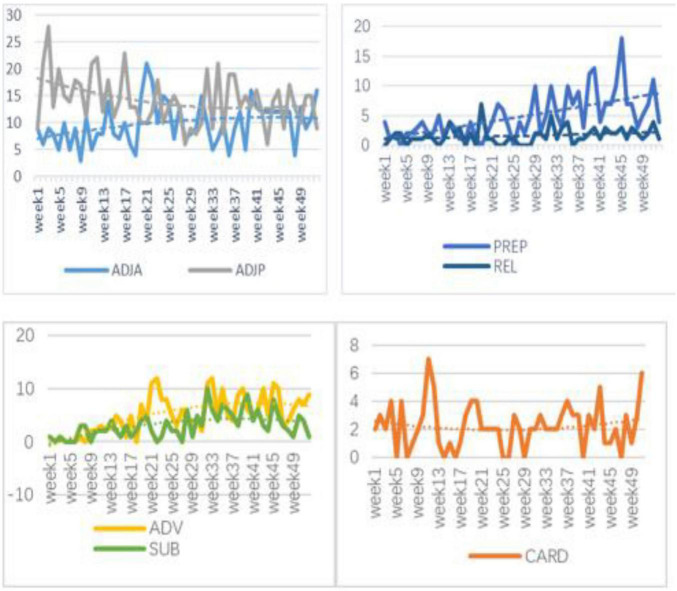
The development of seven fine-grained measures over time.

The data reveal that different modifiers showed different development trends (see Figure 3). ADJA, PREP, and REL showed an upward trend; ADJP showed a downward trend; ADV and SUB showed an upward trend and then a downward trend; and CARD showed no obvious development trend. During the observation period, a competitive relation occurs between ADJA and ADJP subsystems. That is, the growth of ADJA is usually accompanied by a decrease in the usage of ADJP.

### Correlation analysis

Correlation analysis was conducted to investigate the relationship between each two indicators of fine-grained measures, plus between the large-grained and fine-grained indicators.

There is a significant high positive relationship among PREP, MLS, and MLC (*r*_prep–mls__=__0_._738_, *_P_*_<__0_._05;_ *r*_prep–mlt__=__0_._705_, *_*P*_*_<__0_._05_). There is a moderate positive relationship among SUB, MLS and MLC (*r*_sub–mls__=__0_._543_, *_ P_*_<__0_._05;_ *r*_prep–mlt__=__0_._544_, *_*P*_*_<__0_._05_). PREP and SUB were found to be moderately correlated with DC/T and C/T (*r*_prep–dc/t__=__0_._604_, *_ P_*_<__0_._05;_ *r*_prep–c/t__=__0_._616_, *_*P*_*_<__0_._05;_ *r*_sub–dc/t__=__0_._654_, *_*P*_*_<__0_._05;_ *r*_sub–c/t__=__0_._580_, *_*P*_*_<__0_._05_). There is a significant negative relationship among ADJP, MLC and CN/C (*r*_mlc–adjp__=__–0_._288_, *_*P*_*_<__0_._05;_ *r*_cn/c–adjp__=__–0_._324_, *_*P*_*_<__0_._05_).

The correlation results indicate that the increase in the length of sentences and of the T-units takes place not only through expansion at the phrasal level *via* the use of prepositional phrases but also through expansion at the clausal level *via* adverbial clauses by adding adjuncts (of time, place, etc.). When the participant used more Subject + verb + predicative structures (SVP) simple sentence structures, the MLSs, T-units, and clauses tended to decrease, and noun phrases were rarely used. Based on the above correlation analysis, it was found that both the global measures of syntactic complexity, MLS, MLT, DC/T, and CN/C, and the more specific indices of syntactic modifiers, ADJP, ADJA, ADV, SUB, and PREP, can capture the syntactic development changes of the beginner level learners.

## Discussion and conclusion

The indicators of large-grained and fine-grained measures in the writing development were found to be interrelated and affected the coordination and competition in the development process, highlighting the dynamic non-linear and unpredictable complexity characteristics of writing development. In the following discussion, I consider the accidental events that affect the development of the variation process in the non-linear development paths through the content recorded in the data, so as to understand the factors that influence the non-linear developmental trajectory.

According to the findings of Jiang et al. (2019), there are significant differences between MLS, MLT, DC/T in the writing of beginners and intermediate English learners. Hence, I observed these three measures carefully. According to the polynomial trend line, the three measures showed an obvious upward trend. The MLS shows four consecutive peaks (week 29, week 32, week 45, and week 51) and MLT was closely related to the change in MLS. On reading the diary entries for week 29, I found that Alice had gone on a trip with her family during the summer vacation. She described what she saw and felt on the way to the destination. In week 32, Alice attended badminton training class, and described her experience during the game (i.e., how to play well under the guidance of the coach). In week 51, when Christmas, New Year’s Day, and her mother’s birthday were approaching, Alice described in detail how she prepared birthday gifts and how much she looked forward to Christmas and New Year holiday. In this study, an increase in the length of production evident from Alice’s diary is indicative of an obvious increase due to a vacation that was full of rich and colorful activities, as compared to other periods of routine daily life. The result is not consistent with the investigation of Chinese college students’ English writing development by Hou and Chen (2019), which found that the length of production in college students English writing decreased significantly due to the absence of inputs provided during the English classes through winter and summer vacations. Piaget (1997) emphasized the relationship between children’s language development and their active experiences in his theory of cognitive development. Children’s language development is the product of the interaction between their innate psychological cognitive ability and objective experience. Owing to the interaction between Alice’s own life experience and her psychological cognitive ability, it is directly encoded into her thinking and is transformed from experience into word representations; thus the sentence expression is enriched, and an increase in the length of sentences and clauses can be observed.

C/T and MLS reached the highest peak in week 45. While reading the corpus of the 45th week, I found that Alice wrote one diary titled *Money*, listing specific examples of daily consumption of her peers and expressing her disapproval of excessive spending and high consumption. She used subordination, coordination, more complex syntactic structures such as a preposition + *what*-clause in this entry, which is different from other daily narrative entries without topic in the corpus and is reflective of her attempt to state her stand with regard to daily expenses, while discussing and asserting her viewpoint supplemented with examples (Refer to diary examples in Supplementary material). Lu (2011) found the syntactic complexity in argumentative essays which needed more causal reasoning are higher than that in narrative essays. Seven indicators besides C/T and MLS were significantly higher in the data, verifying that genre differences causing differences in syntactic complexity can explain to the writing performance of young beginner learners as well. This is also in line with the results reported by Yang et al. (2015), who found that topic had a significant effect on syntactic complexity features.

The changes in T/S and MLC were almost negligible as compared to MLS and MLT. As a beginner English learner, Alice’s classroom English learning in elementary school was restricted to simple sentences, with no practice of clauses, leading to a negligible increase in MLC and T/S. The input provided plays a key role in the development of language of the learners. During the 1-year observation period, slight fluctuations in the MLC were observed at several instances from a relatively stable state. When MLC becomes relatively stable with little internal variation, it is called an *attractor states* in the DST which refers to states of less variation (de Bot et al., 2005). At this moment more energy is needed to push it out of the attractor states by perturbations outside, such as input in class, reading, immersion in English environment, etc. Although Chinese students start English learning at the primary school, the English curriculum is actually limited to the alphabet and simple daily communication. They are not required to do writing work as part of school learning. Furthermore, limited teaching hours are devoted to English classes in the weekly teaching schedule and there is very limited access to the target language outside the classroom. These contexts are referred to as “low input level” contexts. In case of insufficient input, the MLC system failed to get out of the attractor states with fluctuations; thus, the whole development appeared stable.

Hinkel (2002) found that high frequency of use of predicate adjectives was related to lower second language proficiency, while high frequency of attributive adjectives was related to higher second language proficiency. As evident from Figure 3, the frequency of ADJP in learners’ corpus ranked higher than the other fine-grained indicators but decreased gradually over time, while the ADJA increased significantly, indicating that the participant’s usage of basic English syntactic structures was reduced while more varied syntactic structures were gradually increasing in the dynamic developmental process. The inverse relationship was further observed between ADJP and ADJA over time. That is, when the adjective modifiers increased in the learner’s writing, the predicative adjectives or adverbial adjectives decreased. This is indicative of the interaction in the dynamic systems: Some subsystems support each other while others compete for limited resources, and the development of one leads to the retreat of the other (Van Geert, 1991).

The frequency of both PREP and REL increased as in the case of ADJA. The PREP curves were steeper and exhibited an early rise, and prepositional phrases were found to be used more frequently. REL rose very slowly due to the absence of corresponding classroom input of relative clauses in the elementary school in China.

The frequency of ADV adverb usage was almost zero in the first 6 weeks but increased significantly with the passage of time. I conducted qualitative screening of the adverbs in the corpus, and found that the participant used the common adverbial words as intensifiers very frequently like *really*, *very*, *quite* (*really* is the most common adverb in the corpus), adverbs of frequency like *always*, adverbs of time like *now*, *today*, and *yesterday*, and adverbs of place like *here*, *there*, *home*, and *upstairs*, and conjunction adverbs like *but*, while sentence adverbs like *occasionally*, *actually*, and *alone* were rarely used. It can also be seen that the participant took some modifiers as schematic or fixed chunks, such as “go *back* home,” “*really* good,” “*a bit* tired,” “*a lot* better,” and “*very* funny,” and thus adverb usage lacked diversity. The use of adverbs is a strong discriminator of different proficiency levels or indicator of language development, for which reason Jiang et al. (2019) suggest adding adverb modifiers to the fine-grained indicators to investigate the characteristics of linguistic development, because even advanced English learners are obviously less likely to use adverbs than native speakers.

Cardinal words, having a function similar to ADJA in grammar and playing a role in modifying and defining nouns, show a steady development trend with little variation in the corpus. Numerals are mostly used in descriptive writing such as science and technology. Therefore, the variation can be seen from the polynomial trend that the frequency of numerals changed little over the whole observation period.

Taken together, the developmental process of the young leaner is inevitably accompanied with variability and interplay in terms of subsystems in syntactic complexity. Although the present study has provided us insight in L2 writing developmental paths of one particular learner, the generalizations cannot be extended to all EFL beginner learners. The investigation of Alice’s 1-year English writing development has several practical implications for L2 instructions. First, input plays a significant role in the learner’s language development, and rich life experience can be encoded into young learners’ thinking from experience into words or structures representation, which is more conducive to enriching syntactic expressions during the time of less input (e.g., vacations and holidays) compared to college learners. Second, the findings are in line with other previous research that different genres and topics can cause differences in syntactic complexity, which indicates that even beginner learners are also needed to be assigned a certain genre or topic in writing practice so as to elicit their usage of more syntactic structures. Besides, syntactic modifiers are used in beginner young learners’ syntax in the written output, which should be carefully observed and addressed in their syntactic complexity development by the instructors. Encouragement and targeted feedback can be given to the young learners to help them develop syntactic expressions to express more complex meanings.

## Data availability statement

The data supporting the conclusions of this article are included in the article/Supplementary material, further inquiries can be directed to the corresponding author.

## Ethics statement

Ethical review and approval was not required for the study on human participants in accordance with the local legislation and institutional requirements. Written informed consent to participate in this study was provided by the participants’ legal guardian/next of kin.

## Author contributions

ZW: research design, data acquisition and analysis, interpretation, drafting, and revising the manuscript.
