# Supplementary material for: Dynamic development of syntactic complexity in second language writing: A longitudinal case study of a young Chinese EFL learner
Source: Front Psychol. 2022 Aug 9;13:974481. doi: 10.3389/fpsyg.2022.974481 (PMC9396378; doi:10.3389/fpsyg.2022.974481)
Supplement: Supplementary file 1 [file Data_Sheet_1.docx]

**Supplementary table 1 Fine-grained measures and examples chosen from the corpus**

| Measures | Code | Examples |
| --- | --- | --- |
| Lexical modifiers | ADJA | a *new* gift  my *second* sister |
|  | CARD | I have *two* sisters. |
|  | ADJP | It is *colorful* and *beautiful*.  I am *very happy*. |
|  | ADV | She *really* likes to buy things. |
| Phrasal modifiers | PREP | Dance *in the talent show*.  A way *of teaching* |
| Subordinate clause modifier | SUB | *Although* I will be very tired…  *If* you get right at the riddle*…* |
|  | REL | It is a place *that sells good cars.* |

**Supplementary table 2 Correlation analysis between each indicators of fine-grained measures &between large-grained measures and fine-grained measures**

|  | ADJA | CARD | ADJP | ADV | PREP | SUB | REL |
| --- | --- | --- | --- | --- | --- | --- | --- |
| ADJA | 1.000 | -0.093 | -0.307 | 0.414 | 0.193 | 0.020 | -0.064 |
| CARD | -0.093 | 1.000 | 0.057 | 0.105 | -0.053 | 0.006 | 0.042 |
| ADJP | -0.307 | 0.057 | 1.000 | -0.313 | -0.060 | -0.134 | 0.026 |
| ADV | 0.414 | 0.105 | -0.313 | 1.000 | 0.540 | 0.475 | 0.261 |
| PREP | 0.193 | -0.053 | -0.060 | 0.540 | 1.000 | 0.615 | 0.270 |
| SUB | 0.020 | 0.006 | -0.134 | 0.475 | 0.615 | 1.000 | 0.424 |
| REL | -0.064 | 0.042 | 0.026 | 0.261 | 0.270 | 0.424 | 1.000 |
| MLS | 0.105 | 0.130 | -0.077 | 0.511 | 0.738 | 0.543 | 0.460 |
| MLT | 0.101 | 0.074 | -0.150 | 0.480 | 0.705 | 0.544 | 0.471 |
| MLC | 0.290 | 0.081 | -0.288 | 0.397 | 0.411 | 0.175 | 0.127 |
| T/S | 0.064 | 0.201 | 0.110 | 0.350 | 0.459 | 0.284 | 0.232 |
| DC/T | 0.001 | -0.018 | -0.131 | 0.474 | 0.604 | 0.654 | 0.577 |
| C/T | -0.066 | 0.051 | 0.004 | 0.350 | 0.616 | 0.580 | 0.520 |
| CP/C | 0.021 | -0.143 | -0.230 | -0.042 | -0.216 | -0.191 | 0.089 |
| CN/C | 0.407 | -0.013 | -0.324 | 0.442 | 0.346 | 0.170 | 0.327 |

Note: According to Wolfe-Quintero et al. (1998), correlation greater than 0.650 is a strong correlation, 0.450-0.650 is a moderate correlation, less than 0.450 is a weak correlation, and a negative sign indicates a negative correlation.

**Supplementary table 3 Basic description of corpus**

|  | Total |  | Minimum | Maximum | Mean | Standard Deviation |
| --- | --- | --- | --- | --- | --- | --- |
| Words | 20,665 |  | 189 | 587 | 397.4 | 75.954 |
| Sentences | 2243 |  | 23 | 57 | 43.13 | 7.029 |

**Diary Samples in week 45:**

1. **So** as a student, we should be careful **about what** we are going to buy with our money, **because** it is not easy for parents to earn money, (which) won’t make you feel good, nor others.
2. My other friend Ally, is a girl **who** really likes to buy things. She always spends a lot of money buying toys and art supplies, **but** it’s (they’re) not useful at all.
